# mRNA vaccine encoding Gn provides protection against severe fever with thrombocytopenia syndrome virus in mice

**DOI:** 10.1038/s41541-023-00771-2

**Published:** 2023-10-31

**Authors:** Jae-Yong Kim, Kyeongseok Jeon, Sang-In Park, Yoo-Jin Bang, Hyeong-Jun Park, Hye Won Kwak, Do-Hyung Kim, Soo-Yeon Lee, Eun-Jin Choi, Nam-Hyuk Cho, Jae-Hwan Nam

**Affiliations:** 1 Department of Medical and Biological Sciences, The Catholic University of Korea, Bucheon-si, Gyeonggi-do Republic of Korea; 2 SML Biopharm, Gwangmyeong-si, Gyeonggi-do Republic of Korea; 3 Department of Biomedical Sciences, Seoul National University College of Medicine, Seoul, Republic of Korea; 4 Seoul National University Bundang Hospital, Seongnam-si, Gyeonggi-do Republic of Korea

**Keywords:** Viral infection, RNA vaccines, RNA vaccines

## Abstract

We developed a promising mRNA vaccine against severe fever with thrombocytopenia syndrome (SFTS), an infectious disease caused by the SFTS virus that is primarily transmitted through tick bites. Administration of lipid nanoparticle-encapsulated mRNA-Gn successfully induced neutralizing antibodies and T-cell responses in mice. The vaccinated mice were protected against a lethal SFTS virus challenge, suggesting that this mRNA vaccine may be an effective and successful SFTS vaccine candidate.

Severe fever with thrombocytopenia syndrome (SFTS) is a tick-borne infectious disease that is primarily endemic to China, Japan, and South Korea^1^. The symptoms of this disease include severe fever, thrombocytopenia, vomiting, and diarrhoea. Severe cases may involve multiple organ failure leading to death, with a fatality rate exceeding 10%^1^. SFTS is caused by the SFTS virus (SFTSV), which belongs to the *Bandavirus* genus, *Phenuiviridae* family, and *Bunyavirales* order, and has been officially named Dabie bandavirus^2^. The SFTSV genome is a single-stranded negative-sense RNA comprising three genomic segments^3^. The L segment encodes an RNA-dependent RNA polymerase (RdRp). The M segment encodes glycoproteins (Gn and Gc). A neutralising antibody was shown to bind to a linear epitope in the Gn ectodomain. Therefore, Gn may be a promising vaccine target. The S segment encodes nucleocapsid proteins (NPs) and nonstructural proteins (NSPs)^4–6^.

In this study, we developed a lipid nanoparticle (LNP)-encapsulated mRNA vaccine expressing SFTSV Gn (mRNA-Gn) that effectively induced neutralising antibodies and Gn-specific T-cell responses. Moreover, mice immunised with mRNA-Gn showed perfect protection against lethal SFTSV infection. These results suggest that the SFTSV mRNA vaccine is a promising candidate for protection against SFTSV infections.

The Gn of SFTSV contains neutralising antibody epitopes and potential vaccine targets^5^. Therefore, we developed an mRNA vaccine encoding the SFTSV Gn sequence (mRNA-Gn). The SFTSV Gn sequence (GenBank No. AZN18589.1) was cloned into an mRNA expression vector (PCT/KR2022/020062) (Fig. 1a). To confirm the expression of the SFTSV Gn protein, Vero cells were transfected with mRNA-Gn. The SFTSV Gn protein was detected in cell lysates by western blot analysis (Fig. 1b). We deleted the Tm domain of Gn for expression as the Tm region does not contain any neutralising antibody epitope, and Tm-deleted Gn is better expressed than the complete Gn protein (Supplementary Fig. 1a)^5^.

**Fig. 1:**
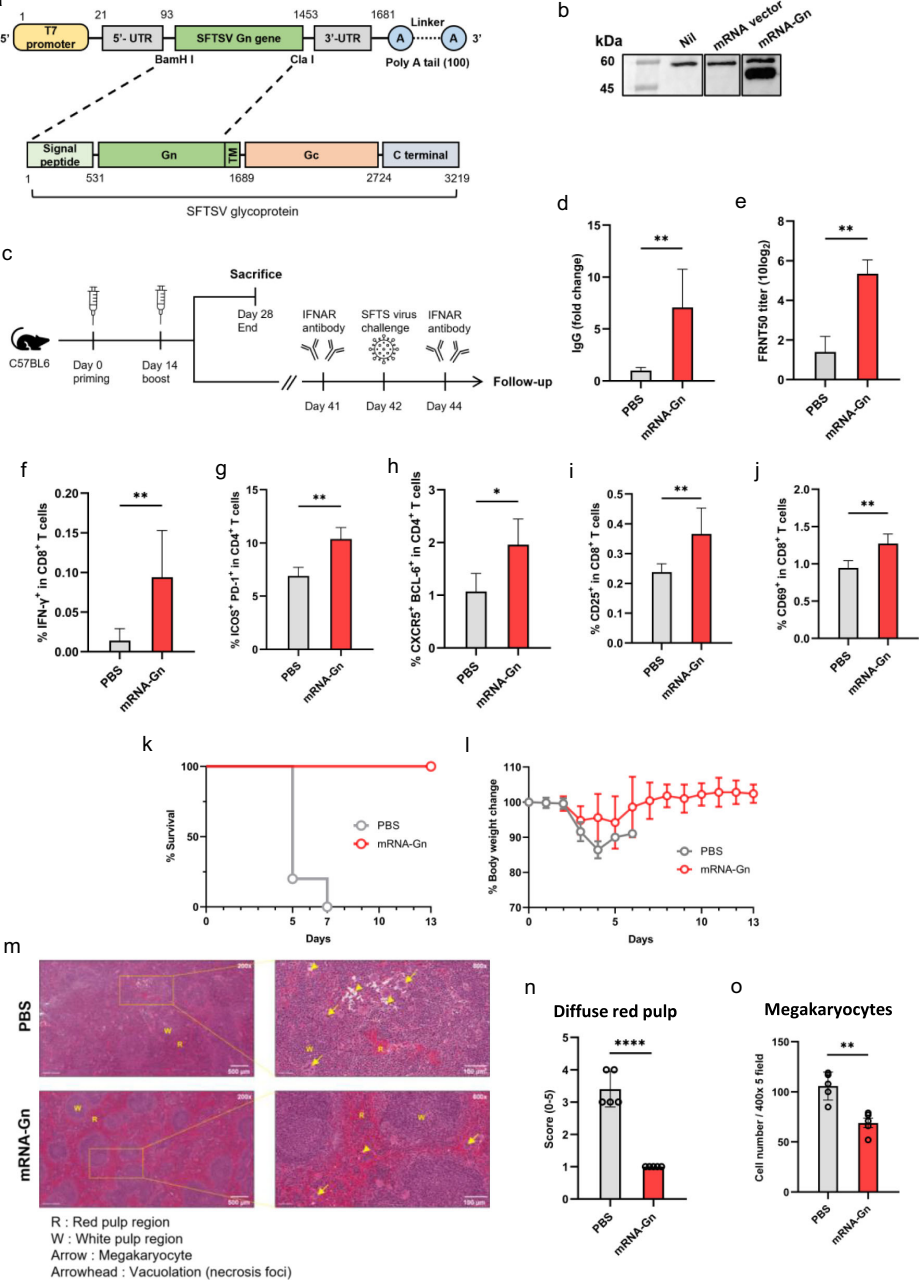
Confirmation of the effective immune responses and protection against SFTSV with mRNA vaccine administration. **a** Structural illustration of the mRNA vaccine encoding the Gn gene of SFTSV. **b** Vero cells were transfected with mRNA-Gn. Western blot analysis showed Gn protein expression. **c** Schematic representation of the experimental design. Two injections were administered at 2-week intervals, and the mice were euthanised 2 weeks after the booster injection for the immunoassay. Ten mice for immunoassays (PBS group: *n* = 5, mRNA-Gn group: *n* = 5) and ten mice for the challenge test (PBS group: *n* = 5, mRNA-Gn group: *n* = 5). **d** The IgG levels in mice were measured, and the fold change was calculated relative to the PBS group. **e** Neutralising antibody titres were measured using the FRNT 50 assay. **f** Proportion of IFN-γ^+^ in CD8^+^ T cells, **g** ICOS^+^ PD-1^+^ in CD4^+^ T cells, **h** CXCR5^+^ BCL-6^+^ in CD4^+^ T cells, **i** CD25^+^ in CD8^+^ T cells, and **j** CD69^+^ in CD8^+^ T cells. The bars represent means ± SD for each group. Statistical analyses were performed using the Mann–Whitney *U* test. (*P < 0.05, **P < 0.01, ***P < 0.001, ****P < 0.001). **k** Survival rates were recorded. **l** Body weight change graph. **m** Pathology of the spleen after virus challenge. Scale bars indicate 500 microns (upper left, lower left) or 100 microns (upper right, lower right). **n** Scoring of the diffuse red pulp in the spleen after virus challenge. **o** Cell number of megakaryocytes in spleen after virus challenge. The bars represent mean ± SD. Statistical analyses were performed using the Mann–Whitney *U* test. (*P < 0.05, **P < 0.01, ***P < 0.001, ****P < 0.001).

To assess vaccine efficacy, 6-week-old C57BL/6 mice were immunised with LNP-encapsulated mRNA-Gn or PBS by intramuscular (IM) injection twice at 14-day intervals. Fourteen days after the boost, the mice were euthanised for further analysis (Fig. 1c). Anti-SFTSV Gn IgG levels significantly increased in the group immunised with mRNA-Gn (Fig. 1d). Neutralising antibodies against SFTSV also showed a significant increase in the group immunised with mRNA-Gn (Fig. 1e). These results indicate that LNP-encapsulated mRNA-Gn elicits a robust humoral immune response against SFTSV.

Next, we evaluated cellular immune responses in mouse splenocytes by analysing Gn-specific T cells using flow cytometry. An increase in the frequency of interferon (IFN)-γ cytokines was observed in CD8^+^ T cells of the mRNA-Gn immunised groups (Fig. 1f). In addition, as follicular helper T (Tfh) cells play an essential role in B-cell maturation, we also analysed Tfh cells^7^. A double-positive plot was created using ICOS and PD-1 markers or CXCR5 and BCL-6 positive plot in the CD4^+^ T-cell population to detect follicular helper T cells. A significantly high frequency of Tfh cells was observed in the mRNA-Gn group (Fig. 1g, h). We also analysed T-cell activation markers, such as CD25 and CD69. The frequencies of CD25^+^ or CD69^+^ markers in CD8^+^ T cells exhibited a comparative increase in the vaccination group. (Fig. 1i, j). These results indicate that mRNA-Gn induces both humoral and cellular immune responses.

An SFTSV challenge test was conducted to evaluate vaccine efficacy. Six-week-old C57BL/6 mice were immunised intramuscularly with LNP-encapsulated mRNA-Gn twice at 14-day intervals. Forty-one days after the boost, an intraperitoneal injection of 100 µg of anti-type I interferon receptor (anti-IFNAR) antibody was administered to the mice 1 day before and 2 days after the SFTSV challenge (Fig. 1c)^8^. After the SFTSV challenge, mRNA-Gn-immunised mice showed protective effects, while mice in all PBS groups died within 7 days (Fig. 1k). mRNA-Gn-immunised mice showed mild weight loss, but recovered their weight 6 days post-challenge (Fig. 1l).

Fourteen days after the viral challenge, histopathological assessments were performed after euthanising the mice. Inflammatory cell infiltration (and ballooning degeneration) and hepatocyte necrosis with shrinking nuclei (or hepatocyte degeneration) in the liver significantly reduced in the mRNA-Gn group than in the phosphate-buffered saline (PBS) group (Supplementary Fig. 2). In particular, the white pulp of the spleen showed mild-to-moderate lymphocyte necrosis and inflammatory foci and an increase in neutrophil and mononuclear cell infiltration because SFTSV targets immature B cells (Fig. 1m)^4,9^. Diffuse red pulp by white pulp atrophy and megakaryocyte formation by a secondary haematopoietic organ were significantly greater in the PBS group than in the mRNA-Gn group (Fig. 1n, o). After the viral challenge, the red pulp of the spleen in the group that received mRNA-Gn showed significantly less expansion than that in the group that received PBS. Since external infection constitutes one of the reasons for the increase in red pulp, this result indicated that the vaccine was effective in protection against the virus. Furthermore, infection with SFTSV caused a reduction in the platelet count, resulting in an increase in megakaryocytes, which are platelet precursors. Interestingly, the vaccinated group showed a significantly lower number of megakaryocytes than the PBS group, thereby indicating effective protection against the virus. These findings demonstrated the efficacy of this vaccine for the prevention of SFTSV infections.

SFTS occurs every year in China, Japan, and Korea, with many of the patients being elderly; however, an appropriate vaccine has not yet been developed. DNA and adenoviral vector vaccines against SFTSV have been reported previously^10–13^. However, in clinical use, DNA vaccines have limitations related to host cell integration, whereas adenoviral vector vaccines have issues concerning anti-adenovirus pre-immunity^14,15^. Thus, safe and effective SFTSV vaccines are still required. However, mRNA vaccines are free from these issues and offer several advantages, including the ability to stimulate robust neutralising antibodies and T-cell immune responses, rapid production, low cost, and scalability^16^. Our mRNA vaccine demonstrated excellent efficacy against neutralising antibodies and protection against SFTSV. Given the numerous benefits of mRNA vaccines, including their rapid production and scalability, they are a promising target to address future outbreaks of SFTS and other viral diseases. Consequently, mRNA-Gn can be expected to become readily available in the event of an urgent need. However, SFTS is most lethal in individuals aged above 50 years, thereby necessitating the investigation of the efficacy of these vaccines in old-aged mice or ferrets.

## Methods

### mRNA preparation

The DNA template for the mRNA vaccine comprised a DNA fragment encoding the SFTSV Gn protein. The DNA templates were cloned into a plasmid vector with backbone sequence elements (T7 promoter, 5′- and 3′-UTR, 100 nucleotide poly(A) tail) interrupted by a linker (A50LA50, 20 nucleotides) to improve RNA stability and translational efficiency. The DNA was purified, quantified using a spectrophotometer (NanoDrop^TM^, ThermoFisher, Waltham, MA, USA), and transcribed in vitro with an EZ™ T7 High Yield In Vitro Transcription kit (Enzynomics, Daejeon, South Korea), a Cap 1 capping analog (SMARTCAP®, ST PHARM, Seoul, South Korea), and N1-methylpseudouridine-5′-triphosphate (m1ΨTP; TriLink, CA, USA) to replace uridine-5′-triphosphate (UTP). After transcription, RNA was purified by lithium chloride precipitation and dsRNA was eliminated by cellulose-based purification. RNA integrity was assessed using gel electrophoresis, and the concentration, pH, and endotoxin levels of the solution were determined.

### LNP formulation

LNPs were prepared according to a reported protocol. Briefly, all the lipid components were dissolved in ethanol at a molar ratio of 25:25:10:38.5:1.5 (SM-102; 6,6′-trehalose dioleate; 1,2-dioleoyl-sn-glycero-3-phosphoethanolamin (DOPE); butyl lithocholate; and 1,2-dimyristoyl-rac-glycero-3-methoxypolyethylene glycol-2000 (DMG-PEG2000))^17^, and mRNA-Gn-LNPs were dissolved at a charge ratio of N/P = 6 in 50 mM sodium citrate buffer (pH 4) solution. LNPs were formulated using enCELL-Master V2 (ENPARTICLE, Busan, Korea) by mixing the aqueous and organic solutions at a ratio of 3:1 with a total flow rate of 10 mL/min. The solution of LNPs was concentrated by ultrafiltration using the Amicon Ultra centrifugal Filter (UFC9030, Merck Millipore, MA, USA), following the manufacturer’s instructions.

### Western blot analysis

Vero cells (Korea Cell Line Bank, Seoul, Korea) were transfected with 5 μg of mRNA-Gn using Lipofectamine 2000® (Invitrogen, Waltham, MA, USA). After 24 h, cell lysates were separated by sodium dodecyl sulphate-polyacrylamide gel electrophoresis (SDS-PAGE) and transferred onto a nitrocellulose membrane. Membranes were blocked with phosphate-buffered saline (PBS) containing 0.1% Tween-20 and 5% skim milk for 1 h at room temperature. The membrane was then incubated overnight at 4 °C with an anti-SFTSV Gn antibody (1:1000 dilution, NBP2-41156; Novus Biological, Centennial, CO, USA) and anti-beta actin antibody (1:3000 dilution, 3700 S; Cell Signaling Technology, Danvers, MA, USA). The secondary antibodies used were anti-rabbit or anti-mouse antibodies conjugated with horseradish peroxidase (1:5000 dilution; A120-101P or A90-116P; BETHYL, Montgomery, TX, USA). Signals were detected using an ECL substrate solution (ECL-PS100; Donginbio, Seoul, Korea). The uncropped western blot images are shown in Supplementary Fig. 1.

### Mice

Six-week-old female C57BL/6 mice were purchased from Koatech (Pyeongtaek-si, Gyeonggi-do, Republic of Korea). The animals were housed at the SMLbiopharm under specific pathogen-free conditions and a standard light cycle (12-h light/dark cycle). All experimental procedures conducted on animals in this study complied with the ARRIVE guidelines and followed the guidelines of and were approved by the Institutional Animal Care and Use Committee of the SML Biopharm (approval numbers: SML-BP-IACUC-2022-10). The animal facility was fully accredited by the Korea Ministry of Science and ICT (LML 22-707, November 17, 2022). At each defined endpoint (14 days after the boost or 14 days after virus challenge), mice were euthanized by CO_2_ inhalation, and their spleens and livers were harvested for immunological analysis.

### Enzyme-linked immunosorbent assay

The presence of antigen-specific IgG in mouse serum was determined using an enzyme-linked immunosorbent assay (ELISA). In this assay, 96-well plates (32296; SPL, South Korea) were coated with 100 ng of Gn protein per well and incubated at 4 °C overnight. The wells were washed thrice with 200 µL of PBS containing 0.05% Tween-20 (PBS-T) and blocked using 100 µL of blocking buffer (1% bovine serum albumin in PBS) for 1 h at room temperature. The diluted serum samples were added to the wells and incubated for 2 h at room temperature. After incubation, the wells were washed thrice with 200 µL of PBS-T. Horseradish peroxidase (HRP)-conjugated anti-mouse anti-IgG (BETHYL; Montgomery, TX, USA) antibodies diluted 1:5000 in blocking buffer were added and incubated for 1 h at 20 °C. After five washes with PBS-T, tetramethylbenzidine (TMB) substrate (ThermoFisher Scientific, Waltham, MA, USA) was added, and the plates were incubated for 10 min, followed by the addition of 2 N H_2_SO_4_ to terminate the reaction. The optical density (OD) values were measured at 450 nm using a GloMax Explorer microplate reader (Promega, Madison, WI, USA).

### SFTSV preparation

SFTSV (GenBank accession no.: MN329148-MN329150) isolated from a Korean patient with SFTS was propagated in Vero E6 cells (ATCC CRL-1586). The supernatant of the infected cells was harvested five days after infection and stored at −80 °C after filtering with a 0.45-μm syringe. Focus-forming unit (FFU) of SFTSV was determined by plaque assay using methylcellulose media [20]. Briefly, the filtered supernatants were serially diluted and added to a monolayer of Vero E6 cells and incubated for 1 h at 37 °C. Viral supernatants were removed and cells were incubated under an overlay media (DMEM supplemented with 5% FBS and 1% methylcellulose) at 37 °C for 7 days. Cells were fixed with 4% paraformaldehyde (Intron, Seongnam, Republic of Korea) and 100% methanol (Merck, Darmstadt, Germany). The SFTSV foci were detected using rabbit anti-SFTS NP antibody (Abclon) and goat anti-rabbit IgG secondary antibody conjugated with alkaline phosphatase (ThermoFisher Scientific). Viral plaques were visualized by incubation with NBT/BCIP solution (Roche, Mannheim, Germany). All experiments with SFTSV were conducted in the Biosafety Level 3 facility at the Seoul National University and Korea Zoonosis Research Institute of Jeonbuk National University.

### Study approval

Animal experiments were conducted in an Animal Biosafety Level 3 facility at the Seoul National University Hospital and Korea Zoonosis Research Institute of Jeonbuk National University. This study was approved by the Seoul National University Hospital and Jeonbuk National University Institutional Animal Care and Use Committee (SNU-220215-4-3, JBNU2022-062), and was conducted in strict accordance with the recommendations in the National Guidelines for the care and use of laboratory animals.

### Neutralizing antibody assay

To evaluate the neutralising activity of the immunised mice, a focused reduction neutralisation titre (FRNT) assay was performed. Briefly, 0.0001 MOI of SFTSV (2015-JJ01 strain; MN329148-329150 in GenBank) was pre-incubated with serially diluted sera from immunised mice at 4 °C for 1 h. A mixture of the virus and serum was added to a monolayer of Vero E6 cells in a 24-well plate. After incubation for 2 h, the supernatant of the cells was replaced with an overlay medium (Dulbecco’s modified Eagle medium supplemented with 2% fetal bovine serum and 0.8% methylcellulose), and the cells were cultured at 37 °C for 7 days. The cells were fixed with 4% paraformaldehyde (Intron, Seongnam, Republic of Korea) and 100% methanol (Merck, Darmstadt, Germany). SFTSV foci were detected using rabbit anti-SFTS NP antibody and anti-rabbit IgG secondary antibody conjugated with alkaline phosphatase (Thermo Fischer, Waltham, MA, USA). Viral foci were visualised by incubation with the NBT/BCIP solution (Roche, Mannheim, Germany). The percentage of focus reduction was calculated as follows: [(number of foci without antibody) × (number of foci with antibody)]/(number of foci without antibody) × 100. The 50% focal reduction neutralisation titres (FRNT 50) were calculated by nonlinear regression analysis (log[inhibitor] versus normalised response method) using GraphPad Prism Software v5.01 (GraphPad Software; http://www.graphpad.com).

### Flow cytometry

Mouse splenocytes (1 × 10^6^) were stained with fluorochrome-conjugated antibodies combined with a solution of PBS and 0.5% foetal bovine serum for flow cytometry. To stain intracellular cytokines, isolated splenocytes were stimulated with 1 μg/mL of Gn protein for 16 h at 37 °C, and brefeldin A (GolgiPlug; BD Biosciences, Franklin Lakes, NJ, USA) was added 6 h post-stimulation. After incubation for 10 h, splenocytes were blocked using CD16/CD32 (101302; BioLegend, San Diego, CA, USA) for 20 min at 4 °C and then stained with CD4 (100414; BioLegend), CD8 (100734; BioLegend), and the Live/Dead staining kit (L34957; Invitrogen, Waltham, MA, USA) for 30 min at 4 °C in the dark. The stained cells were permeabilised using a Fixation/Permeabilization Solution kit (BD Biosciences) for 1 h at 4 °C in the dark and then stained with IFN-γ (505826; BioLegend) for 30 min at 4 °C in the dark. To evaluate the follicular helper T cells or T cells activation, isolated splenocytes were blocked using CD16/CD32 (101302; BioLegend) for 20 min at 4 °C and then stained with a Live/Dead staining kit (L34957; Invitrogen), anti-mouse CD4 (100414, BioLegend), CXCR5 (145506; BioLegend), ICOS (313518; BioLegend), BCL-6 (Biolegend), PD-1 (135255; BioLegend), CD25 (101904; BioLegend), and CD69 (104514; BioLegend) for 30 min at 4 °C in the dark. After washing, the cells were analysed using a Cytek Aurora flow cytometer (Cytek, Fremont, CA, USA) and the results were interpreted using FCS Express (De Novo Software, Pasadena, CA, USA). The gating strategies are depicted in Supplementary Fig. 3.

### Mice immunisation and SFTSV challenge

C57BL/6 WT mice (Total 20, *n* = 5/group) were intramuscularly immunised twice at 2-week intervals with 10 μg of mRNA-Gn-LNP or 50 μL of PBS. One day prior to and 2 days following SFTSV infection, the mice received intraperitoneal injections of 100 μg of anti-mouse IFNAR antibody (BE0241; BioXcell, Lebanon, NH, USA). The mice were subcutaneously challenged with 1 × 10^5^ FFU of SFTSV (GenBank accession no.: NM329148-MN329150). Survival was monitored for 14 days. All SFTSV challenge experiments were conducted in an Animal Biosafety Level 3 facility at the Jeonbuk National University.

### Histological analysis

Sectioned livers and spleens from experimental mice were submerged in 10% neutral buffered formalin, dehydrated, paraffin-embedded, and sectioned at 4-μm thickness for histological examinations. The histological images were obtained and evaluated using Aperio ImageScope version 12.4 (Leica Biosystems Pathology Imaging, Buffalo Grove, IL, USA). The severity of histological changes was determined using a 5-point score system as follows: 0, no abnormality detected (NAD); (1) minimal; (2) mild; (3) moderate; (4) moderately severe; and (5) severe. The distribution was recorded as focal, multifocal, and diffused. The score of vacuolation reflects necrosis by macrophages, and is accompanied with pigment deposition in the spleen. Megakaryocyte numbers were measured in five fields of ×400 magnification. Recruitment of inflammatory cells, necrosis in the liver (Supplementary Fig. 1), and morphological alterations of the liver and spleen were assessed after H&E staining under a light microscope.

### Statistical analysis

All values were expressed as mean ± standard deviation (SD). Statistical analyses were performed using GraphPad Prism software (GraphPad Software Inc., La Jolla, CA, USA). *P* values were determined using the Mann–Whitney *U* test.

### Reporting summary

Further information on research design is available in the Nature Research Reporting Summary linked to this article.

### Supplementary information

REPORTING SUMMARY

Supplementary Figures

## Data Availability

All data generated or analysed during this study are included in this article.
