# Supplementary material for: mRNA vaccine encoding Gn provides protection against severe fever with thrombocytopenia syndrome virus in mice
Source: NPJ Vaccines. 2023 Oct 31;8:167. doi: 10.1038/s41541-023-00771-2 (PMC10618158; doi:10.1038/s41541-023-00771-2)
Supplement: Supplementary file 1 — REPORTING SUMMARY [file 41541_2023_771_MOESM1_ESM.pdf]

nature portfolio

Corresponding author(s): Jae-Hwan Nam

Last updated by author(s): Aug 22, 2023

Reporting Summary

Nature Portfolio wishes to improve the reproducibility of the work that we publish. This form provides structure for consistency and transparency in reporting. For further information on Nature Portfolio policies, see our Editorial Policies and the Editorial Policy Checklist.

Statistics

For all statistical analyses, confirm that the following items are present in the figure legend, table legend, main text, or Methods section.

n/a	Confirmed	
☐	☒	The exact sample size (*n*) for each experimental group/condition, given as a discrete number and unit of measurement
☐	☒	A statement on whether measurements were taken from distinct samples or whether the same sample was measured repeatedly
☐	☒	The statistical test(s) used AND whether they are one- or two-sided<br>*Only common tests should be described solely by name; describe more complex techniques in the Methods section.*
☒	☐	A description of all covariates tested
☐	☒	A description of any assumptions or corrections, such as tests of normality and adjustment for multiple comparisons
☐	☒	A full description of the statistical parameters including central tendency (e.g. means) or other basic estimates (e.g. regression coefficient) AND variation (e.g. standard deviation) or associated estimates of uncertainty (e.g. confidence intervals)
☒	☐	For null hypothesis testing, the test statistic (e.g. *F*, *t*, *r*) with confidence intervals, effect sizes, degrees of freedom and *P* value noted<br>*Give P values as exact values whenever suitable.*
☒	☐	For Bayesian analysis, information on the choice of priors and Markov chain Monte Carlo settings
☒	☐	For hierarchical and complex designs, identification of the appropriate level for tests and full reporting of outcomes
☒	☐	Estimates of effect sizes (e.g. Cohen's *d*, Pearson's *r*), indicating how they were calculated

Our web collection on statistics for biologists contains articles on many of the points above.

Software and code

Policy information about availability of computer code

Data collection	Flow cytometry data was collected using Cytek Aurora flow cytometer.
Data analysis	All statistical analyses were performed using GraphPad Prism 9. Immunohistochemistry images were obtained using Aperio ImageScope v12.4. Flow cytometry results were interpreted using FCS express.

For manuscripts utilizing custom algorithms or software that are central to the research but not yet described in published literature, software must be made available to editors and reviewers. We strongly encourage code deposition in a community repository (e.g. GitHub). See the Nature Portfolio guidelines for submitting code & software for further information.

Data

Policy information about availability of data

All manuscripts must include a data availability statement. This statement should provide the following information, where applicable:

- Accession codes, unique identifiers, or web links for publicly available datasets
- A description of any restrictions on data availability
- For clinical datasets or third party data, please ensure that the statement adheres to our policy

All data supporting the findings of this study are available within the main manuscript and the supplementary files or provided upon reasonable request.

Research involving human participants, their data, or biological material

Policy information about studies with human participants or human data. See also policy information about sex, gender (identity/presentation), and sexual orientation and race, ethnicity and racism.

Reporting on sex and gender	N/A
Reporting on race, ethnicity, or other socially relevant groupings	N/A
Population characteristics	N/A
Recruitment	N/A
Ethics oversight	N/A

Note that full information on the approval of the study protocol must also be provided in the manuscript.

Field-specific reporting

Please select the one below that is the best fit for your research. If you are not sure, read the appropriate sections before making your selection.

☒ Life sciences ☐ Behavioural & social sciences ☐ Ecological, evolutionary & environmental sciences

For a reference copy of the document with all sections, see nature.com/documents/nr-reporting-summary-flat.pdf

Life sciences study design

All studies must disclose on these points even when the disclosure is negative.

Sample size	Sample size for animal experiments (n=5 per group) was selected based on variance observed in prior experiments of a similar nature, as well as practical considerations.
Data exclusions	No data were excluded from analysis.
Replication	FRNT50 titers were repeated twice to obtain the results.
Randomization	No formal randomization method was used.
Blinding	No blinding performed.

Reporting for specific materials, systems and methods

We require information from authors about some types of materials, experimental systems and methods used in many studies. Here, indicate whether each material, system or method listed is relevant to your study. If you are not sure if a list item applies to your research, read the appropriate section before selecting a response.

Materials & experimental systems

n/a	Involved in the study	
☐	☒	Antibodies
☐	☒	Eukaryotic cell lines
☒	☐	Palaeontology and archaeology
☐	☒	Animals and other organisms
☒	☐	Clinical data
☒	☐	Dual use research of concern
☒	☐	Plants

Methods

n/a	Involved in the study	
☒	☐	ChIP-seq
☐	☒	Flow cytometry
☒	☐	MRI-based neuroimaging

Antibodies

Antibodies used	All antibodies details are stated within manuscript and supplemental materials
Validation	All antibodies were used according to manufacture instructions.

Eukaryotic cell lines

Policy information about cell lines and Sex and Gender in Research

Cell line source(s)	Vero cells were obtained from Korea cell line bank.
Authentication	Cell lines used were not authenticated.
Mycoplasma contamination	Cell lines tested negative for mycoplasma.
Commonly misidentified lines (See ICLAC register)	No commonly misidentified lines were used.

Animals and other research organisms

Policy information about studies involving animals; ARRIVE guidelines recommended for reporting animal research, and Sex and Gender in Research

Laboratory animals	Female, C57BL/6, 6 weeks of age.
Wild animals	No wild animals were used.
Reporting on sex	Female mice were used because male mice exhibit aggressive behavior that might affect results.
Field-collected samples	No field-collected samples were used.
Ethics oversight	Animal studies were reviewed and approved by the Institutional Animal Care and Use Committee of SML Biopharm.

Note that full information on the approval of the study protocol must also be provided in the manuscript.

Flow Cytometry

Plots

Confirm that:

☒ The axis labels state the marker and fluorochrome used (e.g. CD4-FITC).

☒ The axis scales are clearly visible. Include numbers along axes only for bottom left plot of group (a 'group' is an analysis of identical markers).

☒ All plots are contour plots with outliers or pseudocolor plots.

☒ A numerical value for number of cells or percentage (with statistics) is provided.

Methodology

Sample preparation	Splenocytes were obtained by isolating mouse spleen using 40um strainers. The obtained splenocytes, as described in supplemental materials, were seeded at a density of 1×10^6 in 96-well round bottom plates. Cells were stimulated with 1 µg/mL Gn protein. 6 hours after stimulations, cells were resuspended in FACS wash buffer (PBS + 2% FBS) and spun at 1200 rpm for 5 minutes. After removing the supernatant, cells were stained with antibodies specific to each analysis panel according to the manufacturer's instructions.
Instrument	Cytek Aurora flow cytometer
Software	FCS Express (De Novo Software)
Cell population abundance	N/A
Gating strategy	1) SSC vs. FSC gating to exclude debris. 2) FSC-H vs. FSC-A gating to exclude doublets.<br>3) SSC-H vs. Live/Dead™ gating to exclued dead cells. 4) SSC-H vs each stained antibodies to quantify antibody positive cells.<br>The gating strategy details are provided in the supplementary information.

☒ Tick this box to confirm that a figure exemplifying the gating strategy is provided in the Supplementary Information.
