# Supplementary material for: mRNA vaccine encoding Gn provides protection against severe fever with thrombocytopenia syndrome virus in mice
Source: NPJ Vaccines. 2023 Oct 31;8:167. doi: 10.1038/s41541-023-00771-2 (PMC10618158; doi:10.1038/s41541-023-00771-2)
Supplement: Supplementary file 2 — Supplementary Figures [file 41541_2023_771_MOESM2_ESM.pdf]

Supplementary Figures

(a)

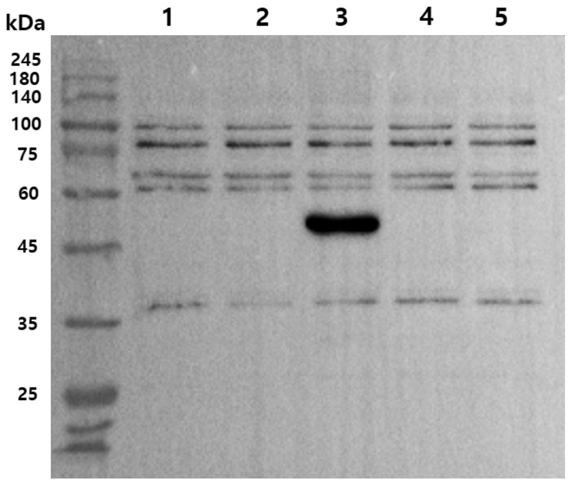

(b)

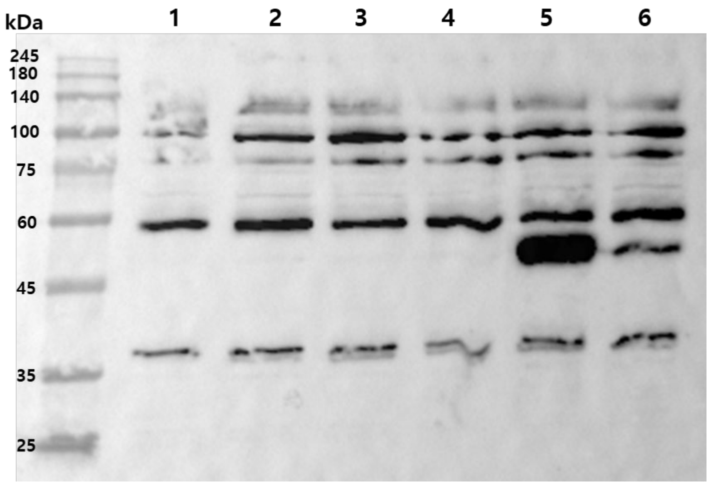

Supplementary figure 1. Uncropped western blot images. (a) Each lane loaded with 10 μg of protein. Lane 1: mRNA-GnGc, Lane 2: mRNA-Gn, Lane 3: mRNA-GnΔTM, Lane 4: mRNA vector, Lane 5: Nil. (b) Uncropped western blot image from Figure 1b. Each lane loaded with 10 μg of protein. Lane 1-2: Nil, Lane 3: mRNA vector (5 μg transfection), Lane 4: mRNA

vector (1 μg transfection), Lane 5: mRNA-GnΔTM (5 μg transfection), Lane 6: mRNA-GnΔTM (1 μg transfection)

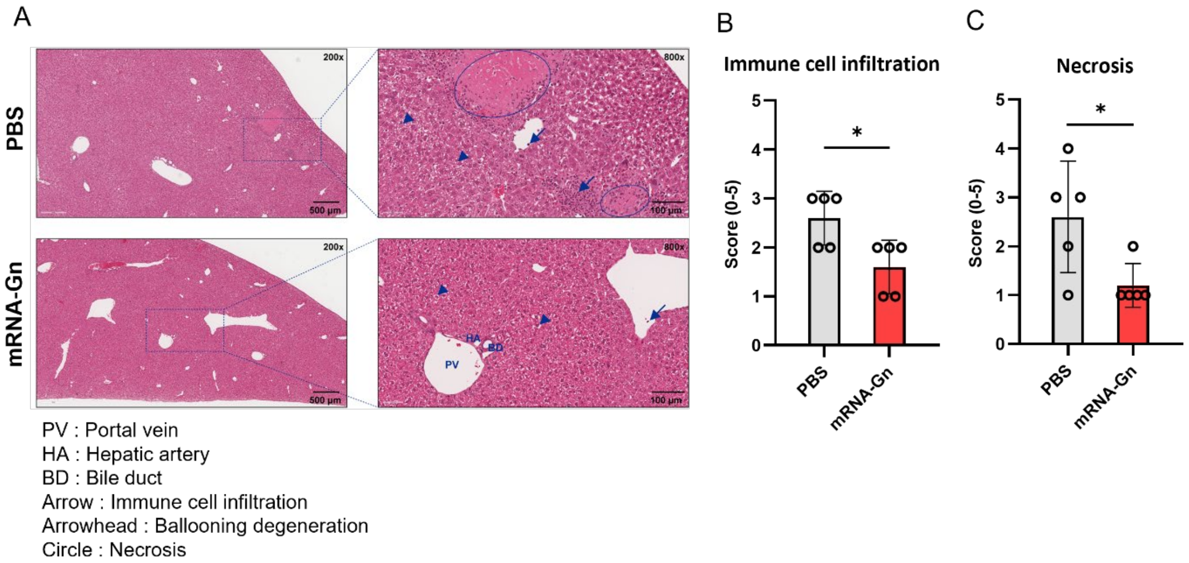

Supplementary figure 2. Pathological findings for the liver after virus challenge. (A) Pathology image of liver. (B, C) Scoring graphs. The bars represent mean ± SD. Statistical analysis was performed using the Mann–Whitney *U* test. (*$p < 0.05$, **$p < 0.01$, ***$p < 0.001$, ****$p < 0.001$)

(a)

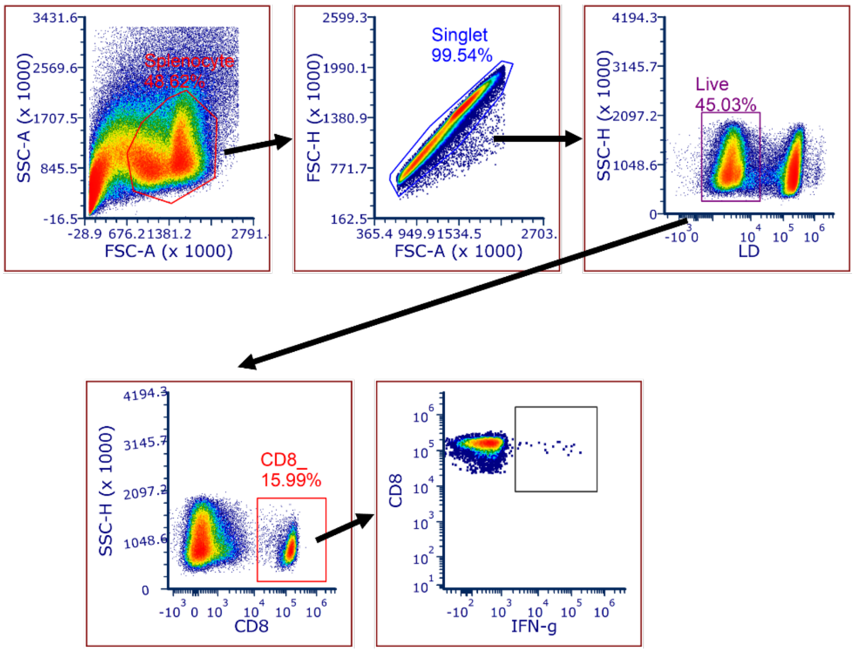
SSC-A (x 1000)
FSC-A (x 1000)
Splenocyte
FSC-H (x 1000)
Singlet
99.54%
SSC-H (x 1000)
Live
45.03%
LD
CD8_
15.99%
CD8
IFN-g

(b)

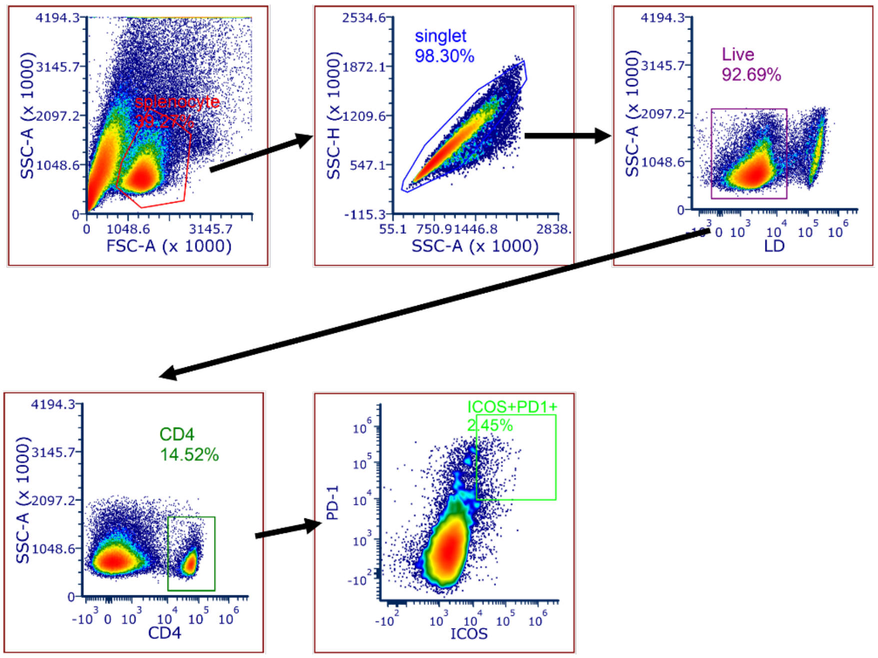
SSC-A (x 1000)
FSC-A (x 1000)
splenocyte
SSC-H (x 1000)
singlet
98.30%
Live
92.69%
LD
CD4
14.52%
ICOS+PD1+
2.45%
PD-1
ICOS

(c)

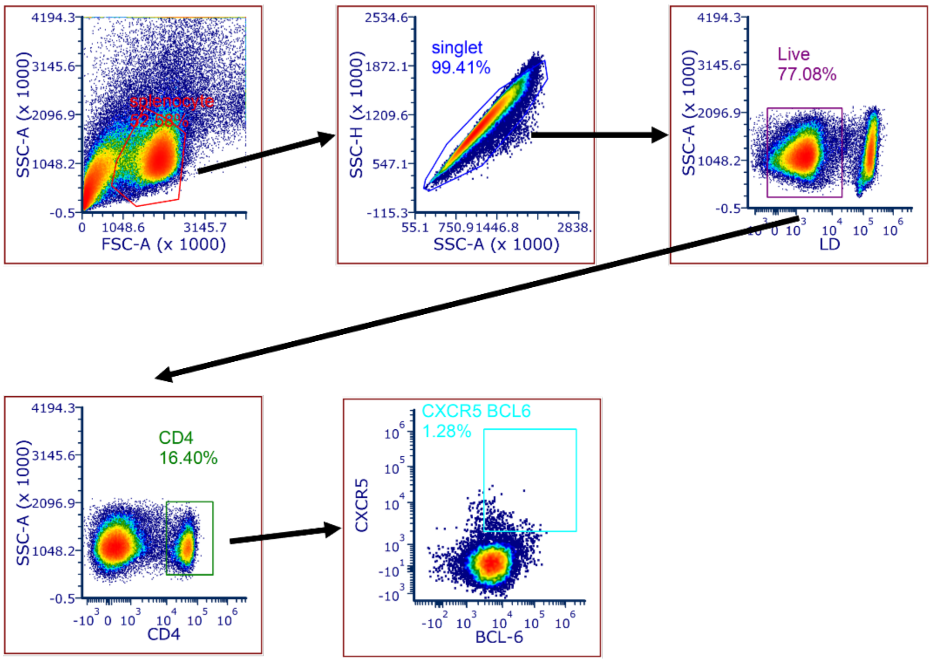

SSC-A (x 1000)
FSC-A (x 1000)
singlet
99.41%
SSC-H (x 1000)
SSC-A (x 1000)
Live
77.08%
LD
CD4
16.40%
CD4
CXCR5 BCL6
1.28%
CXCR5
BCL-6

(d)

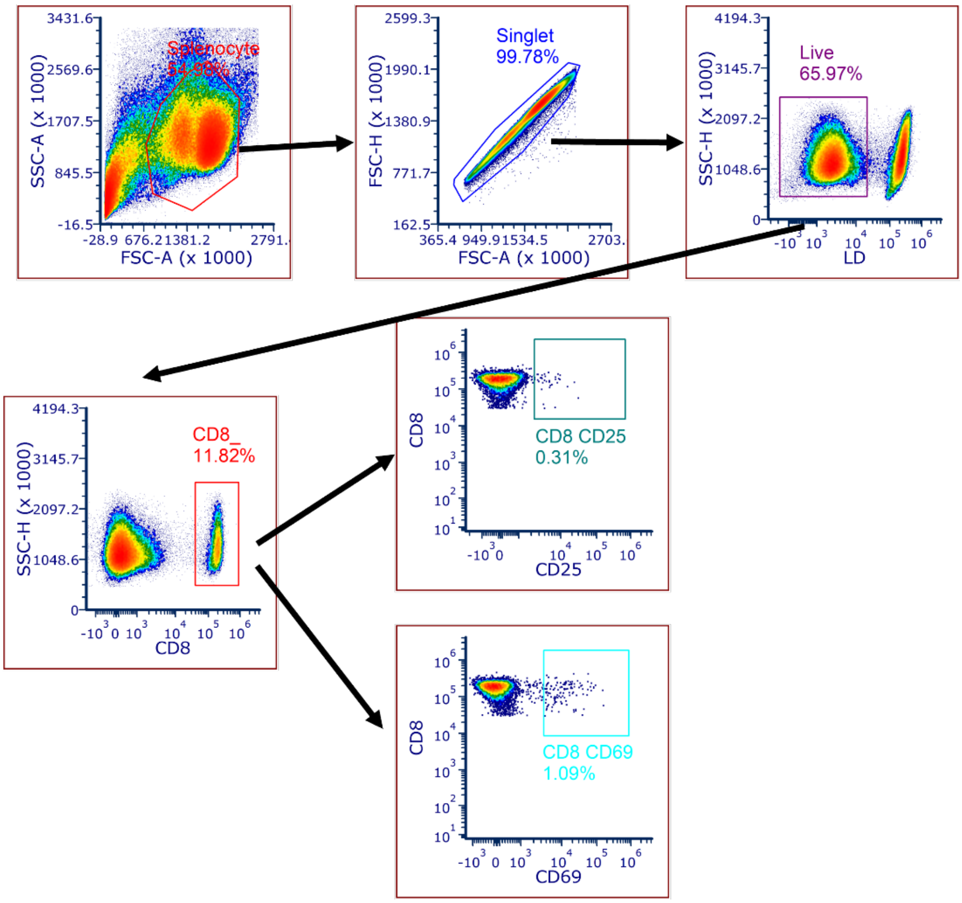

SSC-A (x 1000)
FSC-A (x 1000)
Singlet
99.78%
FSC-H (x 1000)
FSC-A (x 1000)
Live
65.97%
SSC-H (x 1000)
LD
CD8_
11.82%
SSC-H (x 1000)
CD8
CD8 CD25
0.31%
CD8
CD25
CD8 CD69
1.09%
CD8
CD69

Supplementary Figure 3. Flow cytometry gating strategy. (a) IFN-gamma secreting $CD8^+$ T cells, (b) $ICOS^+$ $PD\text{-}1^+$ double positive in $CD4^+$ T cells, (c) $CXCR5^+$ $BCL\text{-}6^+$ double positive in $CD4^+$ T cells and (d) $CD25^+$ or $CD69^+$ in $CD8^+$ T cells.
